# Association of Sarcopenia With Toxicity and Survival in Postoperative Recurrent Esophageal Squamous Cell Carcinoma Patients Receiving Chemoradiotherapy

**DOI:** 10.3389/fonc.2021.655071

**Published:** 2021-07-08

**Authors:** Ying-Ying Xu, Xi-Lei Zhou, Chang-Hua Yu, Wan-Wei Wang, Fu-Zhi Ji, Dong-Cheng He, Wei-Guo Zhu, Yu-Suo Tong

**Affiliations:** Department of Radiation Oncology, Huai’an First People’s Hospital, Nanjing Medical University, Huai’an, China

**Keywords:** sarcopenia, esophageal squamous cell carcinoma, prognosis, chemoradiotherapy, survival

## Abstract

**Background:**

Sarcopenia has been associated with treatment-related toxicities and poor survival in cancer patients. Our aim was to investigate the prevalence of sarcopenia in postoperative recurrent esophageal squamous cell carcinoma (ESCC) patients receiving chemoradiotherapy (CRT) and evaluate associations with treatment-related toxicity and prognosis.

**Methods:**

One hundred and eighty-four patients with postoperative locoregional recurrent ESCC receiving CRT between January 2014 and December 2016 were included. The skeletal muscle area (SMA) was measured at the third lumbar vertebra level. Sarcopenia was defined as skeletal muscle index (SMI = SMA/height^2^) less than 47.24/cm^2^/m^2^ for men and 36.92/cm^2^/m^2^ for women. Association of sarcopenia with overall survival (OS) was analyzed using univariate and multivariate cox regression models.

**Results:**

Sarcopenia was observed in 94 of 184 (51.1%) patients. Sarcopenic patients had significantly higher rates of grade 3-4 toxicities compared to those without sarcopenia (36.2% *vs* 21.1%, *p* = 0.034). The survival rate at 12 and 24 months was 36.2% and 3.2% in the sarcopenic patients and 57.8% and 17.8% in the non-sarcopenic patients (*p* < 0.001). Multivariate cox regression analysis showed that sarcopenia was significantly associated with decreased OS (HR = 1.729, 95% CI 1.231-2.428, *p* = 0.002).

**Conclusions:**

Sarcopenia is an independent indicator of poor survival in postoperative locoregional recurrent ESCC patients treated with CRT. Early nutritional interventions before treatment may improve the prognosis.

## Introduction

Esophageal cancer (EC) is one of the most common cancers worldwide and esophageal squamous cell carcinoma (ESCC) accounts for about 70% of all cases (1). ESCC is highly prevalent in China, and approximate 90% of new cases are ESCC (2). Radical esophagectomy with two-field or three-field lymph node dissection is the primary treatment in locally advanced ESCC. However, the survival of patients treated with surgery alone is poor, with 5-year survival rates of only 25%-39% (3, 4). After surgery, 43%-53% of patients develop locoregional recurrence or distant metastasis (5). For these patients, palliative chemotherapy or chemoradiotherapy (CRT) are commonly used to control cancer-related symptoms and prolong survival. Although these approaches have been demonstrated to be effective in around 50% of patients (6), it is also associated with severe hematological and gastrointestinal toxicities. Thus, discovering factors that could predict CRT-related toxicity and survival in ESCC patients are urgent needs.

Sarcopenia is a common geriatric syndrome, which is initially defined by Baumgartner et al. to describe age-related loss of skeletal muscle mass and skeletal muscle strength (7). Age is the main cause, but not the sole cause of sarcopenia. Malnutrition, low levels of physical activity, several chronic diseases and cancer also induce sarcopenia (8). Many studies have demonstrated that systemic inflammation, inadequate energy and protein intake, as well as increased metabolic rate are independent risk factors for sarcopenia (9). These risk factors are prevalent in ESCC. In a recent study by Anandavadivelan P. et al, sarcopenia and sarcopenic obesity was observed in 43% and 14% of EC patients, and the presence of sarcopenic obesity was a risk factor for developing dose limiting toxicity during neoadjuvant chemotherapy (10). Currently, skeletal muscle area (SMA) on abdomen CT imaging at the level of third lumbar vertebra is widely used for detection of sarcopenia, and it has been demonstrated as a reliable method for whole body muscle mass assessment (11). Sarcopenia is frequently seen in patients with advanced tumor. Prior studies have demonstrated that sarcopenia is associated with poorer overall survival (OS) for a number of malignancies, such as pancreatic cancer, renal cell carcinoma, colorectal cancer (12–14). In an Asian study of ESCC patients receiving neoadjuvant CRT, Ozawa Y et al. found that pretherapeutic sarcopenia was significantly correlated with treatment response and worse disease free survival (15). However, its impact on patients with recurrent or metastatic ESCC remains largely unknown. Thus far, only one study investigated sarcopenia in advanced esophagogastric cancer patients and reported that there was no association between sarcopenia and survival and treatment-related toxicity during palliative chemotherapy (16). However, that study comprised a limited number of patients (only 88 patients included) and the majority of patients were adenocarcinoma (83%). The effect of sarcopenia remains unclear in patients with postoperative locoregional recurrent ESCC.

Therefore, the current study aimed to determine the incidence of sarcopenia in patients with postoperative locoregional recurrent ESCC and to evaluate the relationship between sarcopenia on treatment-related toxicity and OS in patients treated with CRT.

## Materials and Methods

### Patients and Study Design

In this retrospective analysis, patient with ESCC who were treated at our institution from January 1, 2014, to December 31, 2016, for locoregional recurrences after surgery were screened. This time period was chosen in order to have adequate follow-up time for analysis of OS. Postoperative recurrences were confirmed by biopsy, CT, and/or positron emission tomography (PET)/-CT fusion scans. Eligible patients were less than 75 years old; Karnofsky performance status (KPS) score ≥ 70; had histopathologically confirmed ESCC; had local postoperative recurrence (anastomotic recurrence and/or locoregional lymph node metastasis); had upper abdominal CT scan within 3 weeks of treatment start. Patients were excluded if they had incomplete resection, were treated with neoadjuvant or postoperative radiotherapy, had distant metastasis (other than metastasis to supraclavicular or celiac lymph node), or with severe comorbidities. The 7th edition of tumor-node-metastasis (TNM) classification for esophageal carcinoma (UICC, 2009) was used to stage the primary disease after surgery. This study was approved by the institutional review board of Huai’an First Hospital. Informed consent was exempted due to the retrospective nature of the study.

### Treatment Details

Radiotherapy: Patients received either 3-dimensional conformal radiation therapy or intensity modulated radiation therapy. Treatment-planning CT scans (16-slice Philips Brilliance Big Bore CT) using intravenous contrast with a slice thickness of 5 mm were performed for all patients in the supine position. In the present study, all patients were treated with involved field radiotherapy. The gross tumor volume (GTV) included the recurrent tumor or the metastatic lymph nodes. The clinical target volume (CTV) was generated by using 0.8-1 cm radial margin and 1.5-2.0 cm longitudinal margins to the GTV. The planning target volume (PTV) was defined as the CTV plus a 0.5 cm margin in all directions. The prescribed dose was 50 to 66Gy (1.8-2.0 Gy/fraction, 5 days a week) for PTV.

Chemotherapy regimens: Most patients received docetaxel and cisplatin (DP) based chemotherapy in our institution. Among them, 71 patients received concurrent chemotherapy comprising docetaxel (25 mg/m^2^) and cisplatin (25 mg/m^2^) weekly for 5-6 weeks. Another 42 patients received docetaxel and cisplatin regimen consisted of 75 mg/m^2^ docetaxel on days 1 and 22, and 25 mg/m^2^ cisplatin on days 1-3 and 22-24. Approximately 4 to 5 weeks after completion of radiotherapy, at least 2 cycles of adjuvant chemotherapy (docetaxel 75 mg/m^2^ on day 1, cisplatin 25 mg/m^2^ on days 1-3) were given to patients who still have sufficient performance status.

From January 2016, patients aged older than 70 years were treated with S-1 (70 mg/m^2^, twice per day, on days 1–14 and days 22–36) based concurrent chemotherapy to avoid the severe adverse events. In these patients, adjuvant chemotherapy with S-1 (70 mg/m^2^, on days 1-21 every 4 weeks) was done after radiotherapy if possible.

### Body Composition Analysis

**Figure 1:**
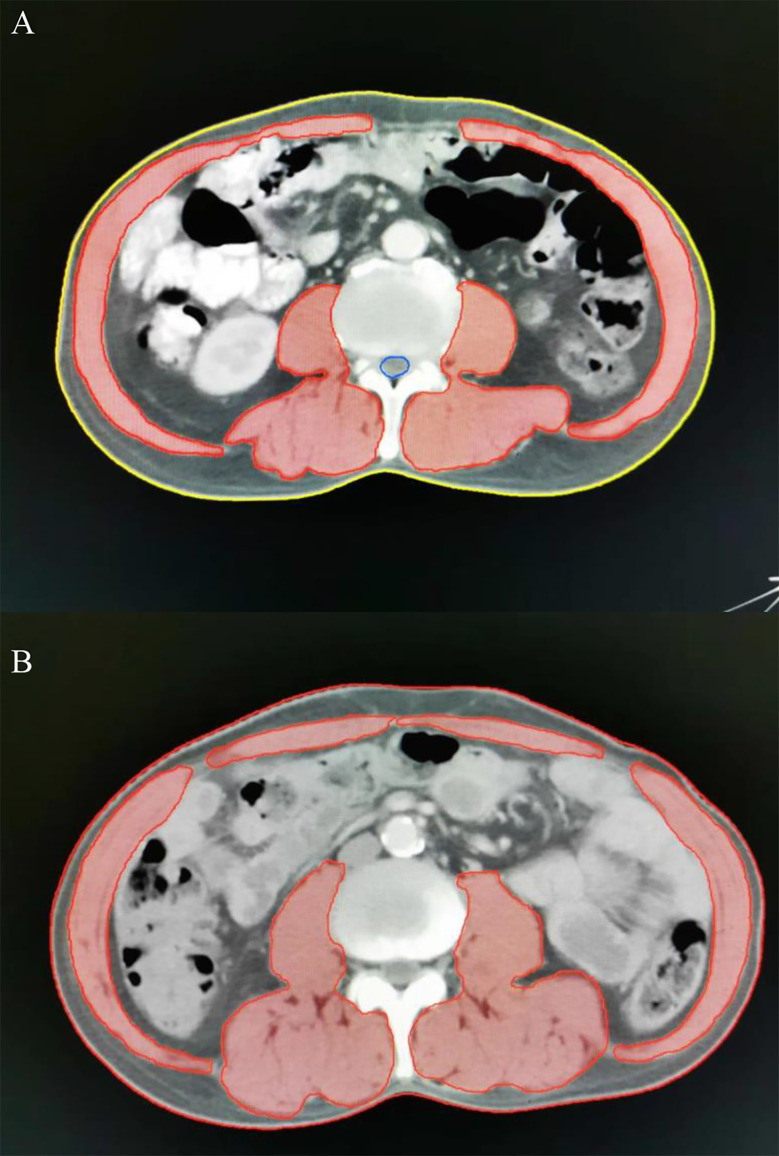
Axial computed tomography (CT) images at the level of L3 with skeletal muscle highlighted in red. Representative CT images in patients with (**A**, a-67-year-old male patient, SMA =122.85 cm^2^, BMI = 21.38 kg/m^2^, SMI=41.04 cm^2^/m^2^) and without sarcopenia (**B**, a-62-year-old male patient, SMA =158.64 cm^2^, BMI = 21.51 kg/m^2^, SMI=56.88 cm^2^/m^2^). SMA, skeletal muscle area; BMI, body mass index; SMI, skeletal muscle index.

Regional muscle tissues were measured by the upper abdominal CT from electronically stored images, which has been done within 3 weeks of radiotherapy. The third lumbar vertebra (L3) was selected as the landmark. Two consecutive CT images extending from L3 were used to measure total muscle cross-sectional area and the mean cross-sectional area (cm^2^) was calculated from each patient. The total SMA including psoas, paraspinal and the abdominal wall muscles (**Figure 1**) were hand-drawn by a senior radiotherapy oncologist. Skeletal muscle was identified and quantified within a Hounsfield unit (HU) range of -29 to +150 HU (17) using the Monaco TPS software (Elekta). The mean cross-sectional areas were normalized to the square of body height (cm^2^/m^2^) and presented as skeletal muscle index (SMI). Sarcopenia was defined as SMI less than 47.24/cm^2^/m^2^ for men and 36.92/cm^2^/m^2^ for women, using previously published cut-off values associated survival in patients with EC (18).

### Patient Data

The present study collected pre-treatment data including patient demographics, serum albumin, weight, height, and body mass index (BMI). The BMI was calculated as weight (kg)/height (m^2^). Underweight, normal weight and overweight were defined as BMI < 18.5, 18.5-24.9, and ≥ 25 kg/m^2^, respectively (19).

### Toxicity Assessment and Follow-up

Toxicity was classified according to the National Cancer Institute Common Toxicity Criteria for Adverse Events (NCI-CTCAE) version 4.0. In the first 2 years after treatment, patients were followed every 3 months, and every 4-6 months thereafter. The final data collection was May, 2020.

### Statistical Analysis

Statistical analysis was performed using SPSS 20.0. Continuous variables are presented as median and range, and categorical variables are presented as number and percentage. For continuous variables, differences between groups were performed using Student’s *t* test or the Mann–Whitney U test. Categorical variables were compared using Chi-square test or Fisher’s exact test. OS was calculated as the time from the last date of radiotherapy to the date of death due to any cause or last follow-up. Data from patients that had not died by the time of analysis were censored. The Kaplan-Meier method was used to determine effects of each variable on OS, and log-rank test was used to compare survival between groups. Univariate and multivariate cox proportional hazards regression models were used to identify prognostic factors of survival. Any factors with *p* value less than 0.10 in the univariate analysis were included in the multivariate analysis. All *p* values were two sided, and level of significance was *p* less than 0.05.

## Results

### Patient and Treatment Characteristics

Between January 1, 2014, and December 31, 2016, 215 patients with postoperative locoregional recurrent ESCC who had received radiotherapy were retrospectively reviewed. Seventeen patients were excluded because the pretreatment CT images were not available for body composition analysis, 14 were excluded due to the lack of complete clinical data. Therefore, a total of 184 patients met inclusion criteria in the final analysis.

Baseline characteristics are displayed in **Table 1**. Of the 184 patients, 141 (76.6%) were male, and the median age was 63 (range, 54-75 years). Almost 90% of patients (166/184) had KPS score ≥ 80. Fifty-seven (31.0%) patients had anastomotic recurrence +/- regional lymph node metastasis, and 127 (69.0%) had only lymph node recurrence (supraclavicular in 28, mediastinal in 39, celiac in 14, and multiregional lymph node metastasis in 46). The diagnosis of recurrence was based on biopsies in 64 patients (34.8%). Follow-up CT and the clinical course were sufficient for diagnosis in the other 120 patients (65.2%). The median time between CT scan and the start of radiotherapy was 7 days (range, 5-21 days).

**Table 1:** Patient and treatment characteristics.

Characteristics	Number (%)
Age (year) median (range)	62 (51-75)
Sex	
Male	141 (76.6%)
Female	43 (23.4%)
KPS	
≥80	166 (90.2%)
70	18 (9.8%)
Primary tumor location	
Upper third	21 (11.4%)
Middle third	124 (67.4%)
Lower third	39 (21.2%)
Stage of primary tumor	
Stage I-II	77 (41.8%)
Stage III	107 (58.2%)
Recurrence pattern	
Anastomosis +/-regional lymph node	57 (31.0%)
Regional lymph node	127 (69.0%)
Treatment	
Radiotherapy alone	20 (10.9%)
CRT	164 (89.1%)
Radiation dose (Gy) median (range)	50.4 (36-66)
Concurrent chemotherapy regimen	
Docetaxel + cisplatin	113 (61.4%)
S-1	51 (27.7%)
Chemotherapy after radiotherapy	
Yes	157 (85.3%)
No	27 (14.7%)
Weight loss in 6 months	
≥ 5%	64 (34.8%)
< 5%	120 (65.2%)
BMI (kg/m^2^)	
< 18.5	36 (19.6%)
18.5-24.9	131 (71.2%)
≥ 25	17 (9.2%)
Albumin (g/L)	
< 35	32 (17.4%)
≥ 35	152 (82.6%)
Diabetes	
Yes	17 (9.2%)
No	167 (90.8%)
SMI (cm^2^/m^2^) median (range)	46.26 (28.62-65.84)

KPS, karnofsky performance status; CRT, chemoradiotherapy; BMI, body mass index; SMI, skeletal muscle index.

All patients were treated with local radiotherapy, and only 4 patients (2.2%) received less than 40Gy because of treatment-related toxicities. Finally, 171 patients (92.9%) completed radiotherapy as planned. The majority of patients (89.1%, 164/184) were treated with concurrent chemotherapy, 113 (61.4%) received concurrent DP and 51 (27.7%) received S-1. Reasons for not received concurrent chemotherapy were KPS score < 80 in 9 patients, refusal in 6, and other reasons in 5.

Before radiotherapy, approximately 34.8% of patients (64/184) had lost ≥ 5% of their body weight in the previous 6 months. The median BMI was 20.35 kg/m^2^ (range, 15.26-27.40 kg/m^2^), and 36 patients (19.6%) were underweight. Overall, the median SMI in all cases was 46.26 cm^2^/m^2^ (range, 28.62-65.84 cm^2^/m^2^).

### Prevalence of and Factors Associated With Sarcopenia

Overall, sarcopenia was found in 94 patients (51.1%). Patients with sarcopenia had worse KPS (*p* = 0.024, **Table 2**) and lower mean BMI (*p* < 0.001, **Table 2**) than those without sarcopenia. Moreover, weight loss ≥ 5% in the previous 6 months (46.8% *versus* 22.2%, *p* = 0.001, **Table 2**) and serum albumin < 35 g/L (23.4% *vs* 11.1%, *p* = 0.033, **Table 2**) were more frequently observed in the sarcopenic patients than in non-sarcopenia patients. With regard to tumor stage and patterns of recurrence, there was no significant difference between the two groups. Patients older than 70 years had a slightly higher prevalence of sarcopenia than those < 70 years (58.5% *vs* 47.1%, *p* = 0.166, **Table 2**), but the difference was not statistically significant.

**Table 2:** Comparisons between patients with and without sarcopenia.

Characteristics	Sarcopenic	Non-sarcopenic	*p*
	n = 94	n = 90	
Age			0.166
< 70	56 (59.6%)	63 (70.0%)	
≥ 70	38 (40.4%)	27 (30.0%)	
Sex			0.384
Male	75 (79.8%)	66 (73.3%)	
Female	19 (20.2%)	24 (26.7%)	
KPS			0.024
≥ 80	80 (85.1%)	86 (95.6%)	
70	14 (14.9%)	4 (4.4%)	
Stage of primary tumor			0.457
Stage I-II	42 (44.7%)	35 (38.9%)	
Stage III	52 (55.3%)	55 (61.1%)	
Recurrence pattern			0.265
Anastomosis	33 (35.1%)	24 (26.7%)	
Regional lymph node	61 (64.9%)	66 (73.3%)	
Diabetes			0.126
Yes	12 (12.8%)	5 (5.6%)	
No	82 (87.2%)	85 (94.4%)	
Weight loss in 6 months			0.001
≥ 5%	44 (46.8%)	20 (22.2%)	
< 5%	50 (53.2%)	70 (77.8%)	
BMI (kg/m^2^), mean (SD)	19.87 ± 2.58	21.15 ± 2.86	< 0.001
< 18.5	25 (26.6%)	11 (12.2%)	0.016
≥ 18.5	69 (73.4%)	79 (87.8%)	
Albumin (g/L)			0.033
< 35	22 (23.4%)	10 (11.1%)	
≥ 35	72 (76.6%)	80 (88.9%)	

KPS, karnofsky performance status; BMI, body mass index.

### Sarcopenia and Treatment-Related Toxicities

Grade 3-4 treatment-related toxicities are shown in **Table 3**. Patients with sarcopenia had significantly higher rates of grade 3-4 toxicities compared to those without sarcopenia (36.2% *vs* 21.1%, *p* = 0.034). The main treatment-related toxicities of grade 3-4 were leukopenia (sarcopenic *vs* non-sarcopenic: 25 [26.6%] *vs* 14 [15.6%], *p* = 0.074), neutropenia (19 [20.2%] *vs* 8 [8.8%], *p* = 0.037), esophagitis (13 [13.8%] *vs* 10 [11.1%], *p* = 0.828), and anorexia (13 [13.8%] *vs* 4 [4.4%], *p* = 0.040).

**Table 3:** Comparisons of treatment-related toxicities between patients with and without sarcopenia.

Toxicities	Sarcopenic	Non-sarcopenic	*p*
	n = 94	n = 90	
Overall toxicity ≥3	34 (36.2%)	19 (21.1%)	0.034
Leukopenia	25 (26.6%)	14 (15.6%)	0.074
Neutropenia	19 (20.2%)	8 (8.8%)	0.037
Anemia	7 (7.4%)	4 (4.4%)	0.537
Thrombocytopenia	4 (4.3%)	5 (5.6%)	0.743
Esophagitis	13 (13.8%)	10 (11.1%)	0.828
Nausea/vomiting	11 (11.7%)	8 (8.9%)	0.631
Anorexia	13 (13.8%)	4 (4.4%)	0.040

With regard to treatment-related death, 2 patients (1 from trachea-esophageal fistula, and 1 from pulmonary embolism) died in the sarcopenic group *versus* 1 (gastrointestinal bleeding) in the non-sarcopenic group.

### Prognostic Significance of Sarcopenia in ESCC Patients

**Figure 2:**
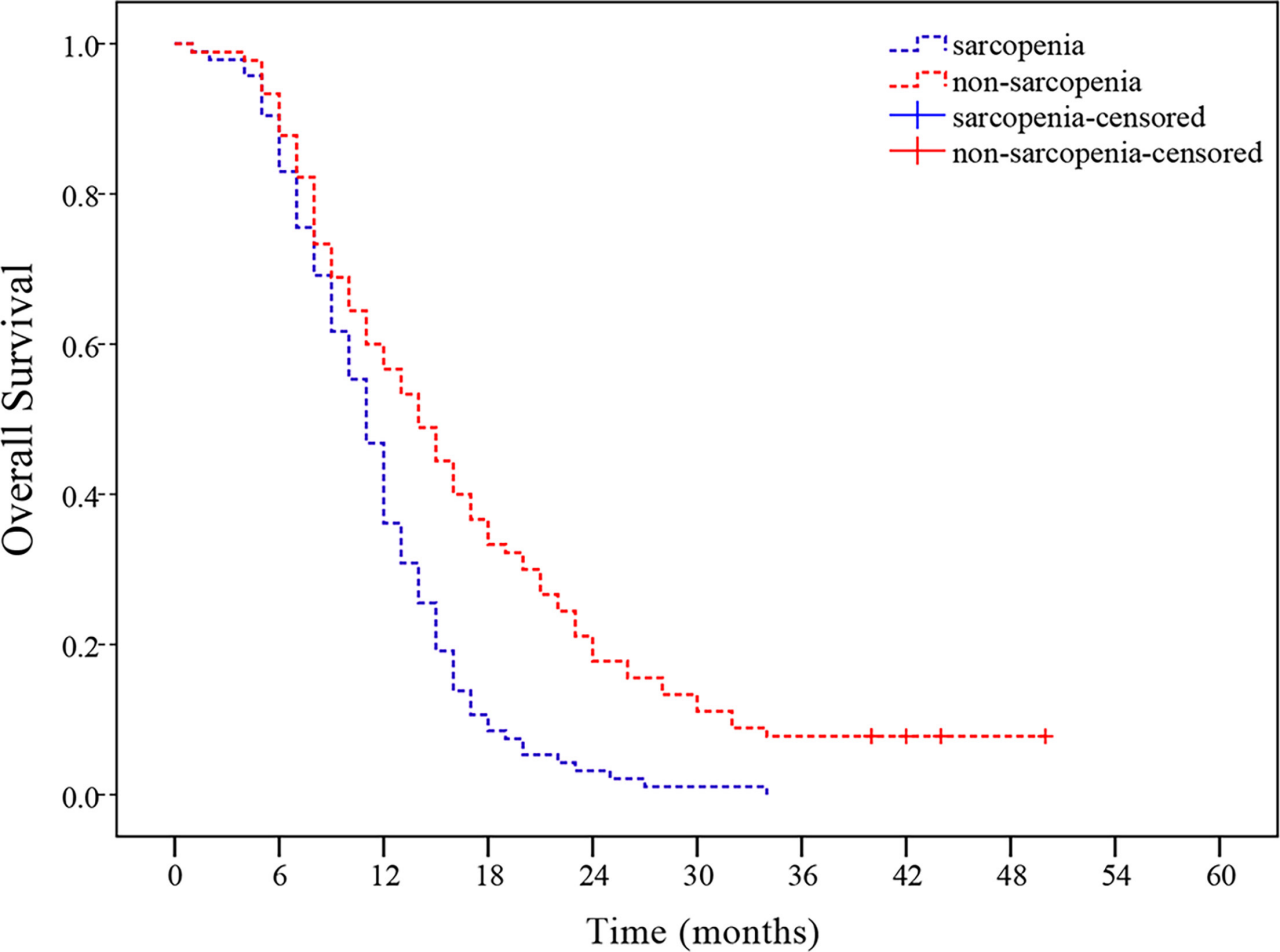
Sarcopenia at presentation and overall survival (OS). Patients with sarcopenia had worse OS than those without sarcopenia (*p* < 0.001).

As of May 18, 2020, median follow-up in this study was 11 months (range, 1-50 months), and 8 patients remained alive at the time of analysis. As observed in the Kaplan-Meier curve, patients with sarcopenia had worse survival compared to those without sarcopenia (*p* < 0.001, **Figure 2**). Kaplan-Meier estimated 12-month OS rate was 36.2% in the sarcopenic patients *versus* 57.8% in the non-sarcopenic patients. The 24-month OS rate for sarcopenic patients was 3.2% compared to 17.8% for non-sarcopenic patients. In univariate analysis, KPS, the use of chemotherapy, weight loss in 6 months, BMI and sarcopenia were significantly associated with poor OS (**Table 4**).

**Table 4:** Univariate and multivariate cox regression analysis for predictors of overall survival.

Variable	Univariate analysis			Multivariate analysis		
	HR	95% CI	*p* value	HR	95% CI	*p* value
Age (years)						
< 70	1.00					
≥ 70	0.788	0.577-1.076	0.134			
Sex						
Female	1.00					
Male	1.331	0.936-1.893	0.112			
KPS						
≥ 80	1.00					
70	2.201	1.342-3.611	0.002	2.331	1.296-4.190	0.005
Primary Tumor location						
Upper third	1.00					
Middle third	0.864	0.539-1.382	0.541			
Lower third	0.962	0.564-1.640	0.887			
Stage of primary tumor						
Stage I-II	1.00					
Stage III	1.313	0.967-1.782	0.081	1.373	1.005-1.874	0.046
Recurrence pattern						
Anastomosis	1.00					
Regional lymph node	1.163	0.846-1.598	0.353			
Concurrent chemotherapy						
Yes	1.00					
No	1.674	1.044-2.683	0.032	1.869	0.721-4.844	0.198
Chemotherapeutic regimen						
Docetaxel + cisplatin	1.00					
S-1	0.833	0.588-1.182	0.307			
Radiation dose (Gy)						
≥ 60	1.00					
< 60	0.768	0.546-1.081	0.130			
Chemotherapy after radiotherapy						
Yes	1.00					
No	1.661	1.094-2.521	0.017	0.633	0.256-1.565	0.322
Weight loss in 6 months						
< 5%	1.00					
≥ 5%	1.404	1.028-1.917	0.033	1.282	0.924-1.779	0.138
BMI (kg/m^2)^						
> 18.5	1.00					
≤ 18.5	1.511	1.041-2.193	0.030	1.455	0.996-2.125	0.053
Baseline albumin (g/L)						
≥ 35	1.00					
< 35	0.993	0.669-1.472	0.970			
SMI						
Non-sarcopenic	1.00					
Sarcopenic	1.907	1.397-2.602	< 0.001	1.729	1.231-2.428	0.002

HR, hazard ratio; CI, confidence interval; KPS, karnofsky performance status; BMI, body mass index; SMI, skeletal muscle index.

Variables with *p* < 0.10 in univariate analysis were included in the multivariate logistic regression analysis. In multivariate model, the presence of sarcopenia was the most significant independent prognostic factor of poor OS (*p* = 0.002) followed by worse KPS and advanced tumor stage (**Table 4**).

## Discussion

It is well established that sarcopenia is a significant factor of poor survival across various cancer types (20–22). However, to our knowledge, there are no reports discussing the relationship between sarcopenia and survival in postoperative locoregional recurrent ESCC patients. Thus, in the present study, we first investigated the incidence of sarcopenia in 184 patients with postoperative locoregional recurrent ESCC and then evaluated associations with treatment toxicity and survival. Our results confirmed that over 50% of patients had sarcopenia. These patients were more likely to present grade ≥ 3 toxicities compared with non-sarcopenic patients. In addition, the multivariable analysis showed that sarcopenia was a significant independent prognostic factor for poor survival.

The prevalence of sarcopenia in patients with ESCC fluctuates significantly, with reports ranging from 16% to 75% (23). However, these studies vary in the definition of sarcopenia, tumor stage and histological type. In this study, inclusion criteria were limited to patient with postoperative locoregional recurrent diseases, and 51.1% (94/184) of patients had sarcopenia at presentation, which is higher than a prior study (16%) involving patients with locally advanced EC (9). However, because the majority of patients (81.2%) in that study were esophageal adenocarcinoma and 43% was visceral obesity, direct comparison with the current study is difficult. To date, the optimal cut-off values chosen to diagnose sarcopenia remain a matter of debate. In western countries, the sex-specific cut-off values for L3 SMI (52.4/cm^2^/m^2^ for men and 38.5/cm^2^/m^2^ for women) proposed by Prado et al. are most commonly used to evaluate sarcopenia in patients with cancer (24). Using the Prado’s criteria, the population of sarcopenia in the present study would increase to 129 (70.1%), which was consistent with that in Siegal SR study in patients with EC (25). However, this criteria might not be applicable to Chinese ESCC patients because the BMI differs considerably between Asian and western populations. In the present population, we diagnosed sarcopenia according to the cut-off values proposed by Nakashima Y (18). As the population of that study is very similar to our population. In ESCC, the nutritional impairment due to dysphagia, pain, systemic inflammation and increased metabolic rate may have promoted the development of sarcopenia.

Several studies have reported that patients with sarcopenia had higher rates of treatment-related toxicity in various malignancies (26). In a study on patients with metastatic breast cancer, chemotherapy toxicity was more commonly observed among sarcopenic patients (27).. In locally advanced EC patients treated with neoadjuvant radiochemotherapy, Panje CM et al. showed that the incidence of grade ≥ 3 toxicity was significantly higher in sarcopenic compared with non-sarcopenic patients (28). In EC patients treated by esophagectomy, Ida S et al. revealed that sarcopenia was closely associated with higher rates of respiratory complications (29). In our study, patients with sarcopenia had greater risk of grade 3-4 CRT-related toxicity, so we believe that the clinical management of patients with sarcopenia before CRT, such as physical exercise, nutrition management, as well as pharmacologic treatment, could prevent toxicity.

Sarcopenia has been correlated with shorter survival in certain solid cancers such as oropharyngeal squamous cell carcinoma, non-small cell lung cancer, and nasopharyngeal carcinoma (30–32). Currently, to our knowledge, 6 studies investigated the effect of sarcopenia on survival in EC patients who received surgical resection (9, 18, 33–36). Three of these studies reported that sarcopenia was significantly associated with poor survival. However, the impact of sarcopenia on survival after CRT has not been clearly established in patients with postoperative locoregional recurrent ESCC. In the current study, we found that the 12-month and 24-month OS were significantly lower for patients with sarcopenia compared to those without, which is consistent with 2 recent studies on patients with unresectable advanced EC treated with CRT (37, 38). In contrast, in another study of 300 patients who treated with trimodality therapy, sarcopenia was not associated with poor OS in a subset of 61 patients who underwent neoadjuvant radiochemotherapy (28). This may be because the numbers of patients were relatively small and the cut-off values used for sarcopenia were looser than ours. Furthermore, differences in treatments and stage of disease may also affect the results.

Our study has several limitations. Firstly, the conclusions are drawn from a sing-institute retrospective analysis and the sample size was small. Secondly, given its retrospective design, evaluation of muscle strength and physical activity were not available. Thirdly, a small proportion of patients (21.7%) received anlotinib-targeted therapy or PD-1 inhibitor after tumor progression. This may have affected our results. Thus, further multi-institutional prospective clinical trials are needed to confirm our results.

In conclusion, our results show that sarcopenia is significantly associated with treatment-related toxicity and poorer outcomes in postoperative locoregional recurrent ESCC patients receiving CRT.

## Data Availability Statement

The original contributions presented in the study are included in the article/supplementary material. Further inquiries can be directed to the corresponding authors.

## Ethics Statement

The studies involving human participants were reviewed and approved by Huai’an First Hospital. Written informed consent for participation was not required for this study in accordance with the national legislation and the institutional requirements.

## Author Contributions

Y-YX, X-LZ, W-GZ, and YT conceived and designed the experiments and were responsible for data analysis and writing the manuscript. C-HY, W-WW, and F-ZJ were responsible for providing the clinical samples. D-CH was responsible for data collection. All authors contributed to the article and approved the submitted version.

## Funding

This work was supported by grants from National Nature Science Foundation of China (Grant No. 82002536) and Huai’an Natural Science Research Project (HAB201930). These funding agencies were not involved in the study design, the collection, analysis and interpretation of data, writing the report, and the decision to submit the article for publication.

## Conflict of Interest

The authors declare that the research was conducted in the absence of any commercial or financial relationships that could be construed as a potential conflict of interest.
